# Impact of a New Portable Air Purification Technology Device in the Pediatric Hospital Setting – A Pre-post Assessment Study

**DOI:** 10.7759/cureus.7440

**Published:** 2020-03-27

**Authors:** Nikhil G Rao, Ambuj Kumar, Chelsea Colon, D. Y Goswami

**Affiliations:** 1 Clinical Trials, Molekule Inc., San Francisco, USA; 2 Internal Medicine, University of South Florida Morsani College of Medicine, Tampa, USA; 3 Pediatrics, Mercy Health, Rockford, USA; 4 Chemical and Biomedical Engineering, University of South Florida, Tampa, USA

**Keywords:** peco, icu, air, pediatrics, length of stay

## Abstract

Introduction

We assessed whether portable photo-electrochemical oxidation (PECO) air purification in the pediatric hospital room setting could improve health outcomes for patients admitted with respiratory distress.

Methods

We performed a prospective study evaluating the use of a portable air purifier with PECO technology. The historical control group comprised matched patients. Twenty-seven PECO-equipped portable air filtration devices were placed in the rooms. Clinical endpoints included length of stay in the hospital, length of stay in the intensive care unit (ICU), rates of intubation, non-invasive ventilation, and nebulizer use.

Results

The mean length of ICU stay was 0.7 days in the pre-intervention period and decreased to 0.4 days post-intervention. The mean length of overall hospitalization reduced by 0.3 days. The rate of non-invasive ventilation use was 77% in the pre-intervention period and decreased to 23% in the post-intervention period. The rate of nebulizer use was 59% in the pre-intervention period and 41% in the post-intervention period. The rate of intubation was 57.1% in the pre-intervention period and 43% in the post-intervention period.

Conclusion

Portable PECO air purification may reduce hospital length of stay, rates of intubation, and need for non-invasive intervention and nebulizers for pediatric patients admitted with respiratory distress.

## Introduction

The quality of the air within the hospital can significantly impact the health of individuals within this space [1]. Specifically, the air provides a vehicle for the spread of bacteria, viruses, fungi, and other airborne toxins and can contribute to potentially lethal hospital-acquired infections [2-4]. Hospitals predominantly rely on high minimum efficiency reporting value (MERV) rated filters to maintain air filtration through the central heating, ventilation, air conditioning (HVAC) system [1,5]. Despite the use of these filters, airborne-related infections continue to be a problem within the hospital setting [6]. Previously, we have described the use of a new air purification technology in the home setting to reduce respiratory allergy symptoms [7]. This technology uses an efficient photo-electrochemical oxidation (PECO) reaction on a nano-coated filter to oxidize and mineralizes chemical and microbiological organic matter included in the air as it passes through the device. Such organic matter includes volatile organic chemicals (VOCs), bacteria, viruses, and fungi [7]. Our objective for this study was to assess whether portable PECO air purification in the pediatric hospital setting could improve health outcomes for patients admitted with respiratory distress. 

## Materials and methods

**Figure 1:**
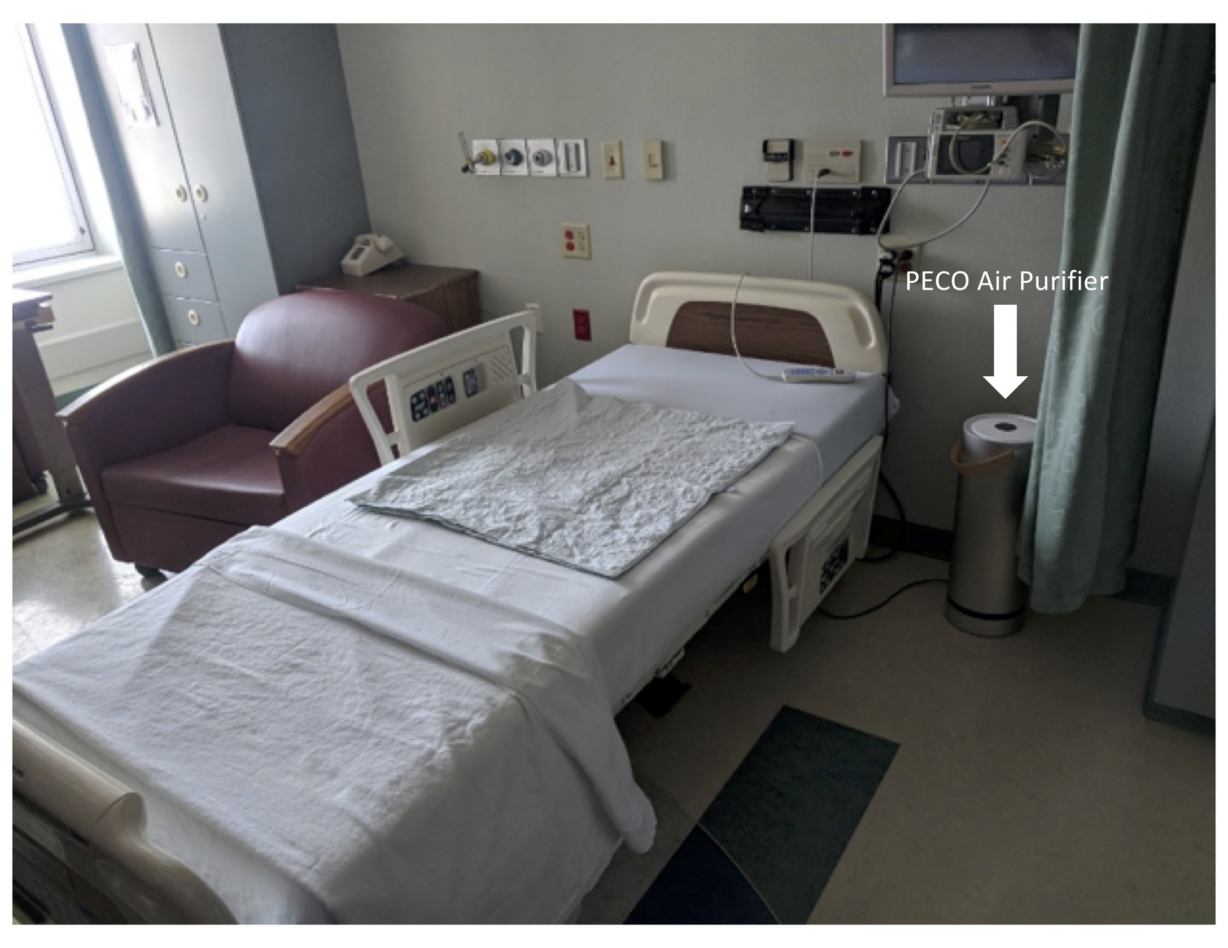
PECO portable air purifier placement in the patient room near the head of the bed PECO, photo-electrochemical oxidation

We performed a prospective non-randomized controlled pre-post study evaluating the use of a portable air purifier with PECO technology in the pediatric ward setting for the reduction of non-invasive ventilation use. The study was performed between August 2018 and December 2018 at one hospital site. All consecutive pediatric patients admitted between August 2017 and August 2018 served as the intervention group, and all consecutive admissions in the year preceding the intervention served as the control. The study was approved by the hospital’s institutional review board and was registered on clinical trials.gov., NCT03647397. The intervention in the study was 27 PECO-equipped portable air filtration devices (MH-1) placed in all pediatric rooms within the hospital in a safe location so as not to disrupt the patient’s routine medical care (Figure 1).

The units were placed as close to the patient’s breathing zone as safely possible with appropriate safety precautions. Units were placed in 20 private rooms and in 7 pediatric intensive care unit (ICU) rooms. Staff members were trained to operate the devices and to ensure that they were running 24 hours a day. If patients were bothered by the unit’s sound or light, staff were trained in turning off the devices. The pediatric patients included in the study were admitted with a diagnosis of infectious or non-infectious respiratory distress during the hospitalization identified by specific ICD codes. These included those with acute respiratory infections, influenza, and pneumonia, other acute lower respiratory infections, other diseases of the upper respiratory tract, chronic lower respiratory diseases, lung diseases due to external agents, other respiratory diseases principally affecting the interstitium, suppurative and necrotic conditions of the lower respiratory tract, other diseases of the pleura and post-procedural complications and disorders of the respiratory system not elsewhere classified. Patients were excluded if they were only in the emergency department or admitted to the neonatal intensive care unit.

A single independent researcher collected the data from the electronic medical records during the intervention and the initial control phase. Another independent biostatistician performed the analysis of the de-identified data set. The primary outcome for the study was the reduction in non-invasive ventilation use. The secondary outcomes were the length of stay in the hospital, length of stay in the ICU, duration of intubation, and nebulizer use. Air filter sampling is discussed in the supplement. 

Statistical methods

Descriptive statistics as frequency and relative percentages were used to summarize categorical data and as means and standard deviations (SD) for continuous data. The adjusted and unadjusted rate of events for primary outcomes in the pre and post-intervention phase was assessed using binary logistic regression. The same approach was used for binary secondary outcomes and summarized as odds ratios along with 95% CI. To adjust for confounders of admitting diagnosis and age, we calculated a propensity score, which was used for all adjusted analysis. The change in outcomes following intervention for continuous variables was assessed using independent samples t-test and summarized as mean differences along with 95% confidence intervals (CI). The statistical significance was set at p<0.05 for all comparisons. All analyses were be performed using SPSS statistical analysis software.

## Results

Altogether 562 patients met the inclusion criteria, of which 273 were admitted in the pre-intervention phase and 289 in the post-intervention phase. As shown in Table 1, the pre- and post-intervention groups were balanced for gender, age, race admitting diagnosis, and asthmatic status. Briefly, 56% of subjects were females in the pre-intervention cohort versus 42% in the post-intervention period (p=0.767). The mean age of patients in the pre-intervention cohort was 4 years (±4) versus 4.4 years in (±4.4) the post-intervention cohort (p=0.078).

**Table 1:** Patient demographic information

	Pre-intervention	Post-intervention	P-value
Variables			
Gender			
Male	58% (228)	42% (168)	0.767
Female	56% (137)	44% (106)	
Age	Mean 4 years (±4.1)	Mean 4.4 (±4.4)	0.078
Race/ethnicity			
White	56% (190)	44% (148)	0.556
African American	57% (96)	43% (73)	
Hispanic	100% (3)	0% (0)	
Asian	60% (6)	40% (4)	
Admitting diagnosis category			
Respiratory	58% (317)	42% (226)	0.321
Non-respiratory	54% (73)	46% (63)	
Asthma as admitting diagnosis			
Mild	50% (13)	50% (13)	0.192
Moderate	49% (21)	51% (22)	
Severe	57% (4)	43% (3)	
Unspecified	66% (61)	34% (31)	

Non-invasive ventilation use

**Figure 2:**
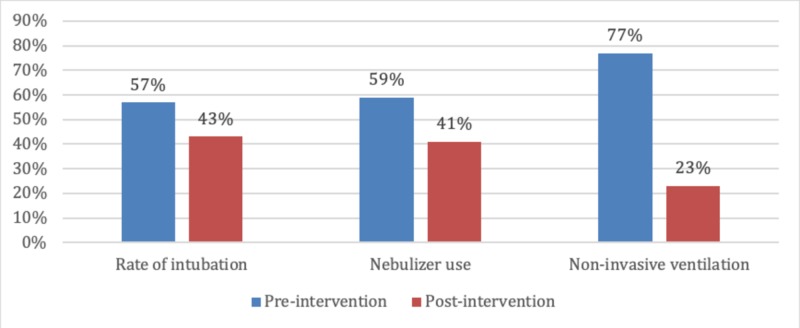
Rates of intubation, nebulizer use and non-invasive ventilation use pre- versus post-intervention

The rate of non-invasive ventilation use was 77% in the pre-intervention period and 23% in the post-intervention period (Figure 2).

The decrease in non-invasive ventilation use in the pre-intervention cohort compared with the post-intervention cohort was statistically not significant. The unadjusted odds ratio was 0.38 (95% CI 0.14 to 1.05; p=0.062). The propensity score-adjusted odds ratio was 0.41 (95% CI 0.15to 1.14; p=0.089). 

Nebulizer use

The rate of nebulizer use was 59% in the pre-intervention period and 41% in the post-intervention period (Figure 2). The decrease in nebulizer use in the pre-intervention cohort compared with the post-intervention cohort was statistically not significant. The unadjusted odds ratio was 0.78 (95% CI 0.56 to 1.09; p=0.150). The propensity score-adjusted odds ratio was 0.84 (95% CI 0.60 to 1.19; p=0.335). 

Rate of intubation

The rate of intubation was 57.1% in the pre-intervention period and 43% in the post-intervention period (p=0.995; Figure 2). The decrease in the rate of intubation in the pre-intervention cohort compared with the post-intervention cohort was statistically not significant. The unadjusted odds ratio was 0.99 (95% CI 0.22 to 4.48; p=0.995). The propensity score-adjusted odds ratio was 0.90 (95% CI 0.22 to 4.0; p=0.897). 

Pediatric ICU stay

**Figure 3:**
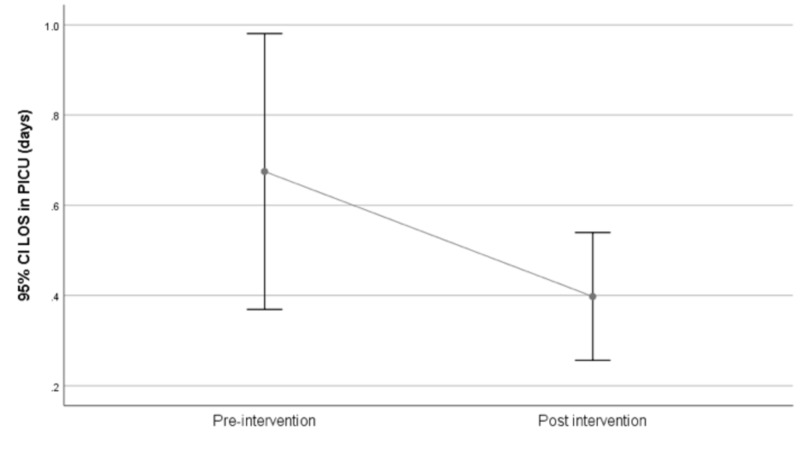
PICU length of stay pre- versus post-intervention PICU, pediatric intensive care unit

The mean length of stay was 0.7 days (±2.9) in the pre-intervention period versus 0.4 days (±1.2) in the post-intervention period, resulting in a mean difference of 0.3 days (95% CI -0.09 to 0.65; p=0.146; Figure 3).

Overall length of hospitalization

**Figure 4:**
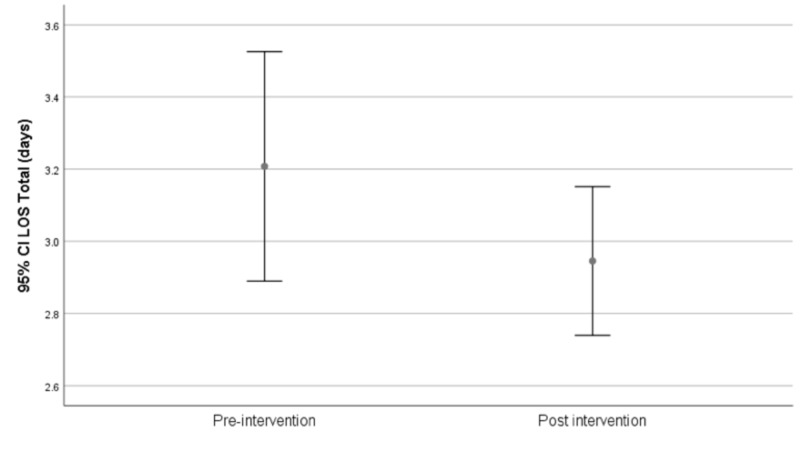
Overall length of hospitalization pre- versus post-intervention

The mean length of overall hospitalization was 3.2 days (± 3) in the pre-intervention period versus 2.9 days (±1.7) in the post-intervention period resulting in a mean difference of 0.26 days (95% CI -0.146 to 0.670; p=0.207; Figure 4).

Adverse events

No adverse events were reported during the study attributable to the portable air purifiers.

## Discussion

The findings show that the use of a portable air purification PECO technology is associated with reductions in overall length of hospital stay, PICU stay, intubation, nebulizer, and non-invasive ventilation use for pediatric patients admitted with respiratory distress. While the results are not statistically significant, it is indeed clinically meaningful with significant impact on the healthcare system. For example, the absolute difference in the use of non-invasive ventilation was 54% which in number need to treat metric translates to a value of 2, meaning that for every two patients, the use of air purification technology prevents 2 additional uses of non-invasive ventilation in comparison to the standard of care.

These reductions can lead to significant cost savings for the hospital from reduced length of stay and needed interventions and may be associated with significant reductions in patient morbidity. The findings from our study show that the hospital central HVAC system and MERV-rated filters can reduce bacteria and fungi in the air to some extent, but bacteria and mold still exist on the final filter before this air enters into the hospital rooms (see supplement). We observed that in the patient’s local environment bacteria, mold and likely other airborne toxins can exist at very high levels. The reasons for this are not entirely clear but may be due to the pathogens or pollutants carried by the patient or hospital workers, those disturbed by movement in the room or otherwise those that are not effectively filtered by the central HVAC system. We show that portable PECO technology is able to reduce bacterial and fungal counts to undetectable levels on the final filter surface. 

Airborne related infections in the hospital can have significant morbidity and mortality in the hospital setting. Existing technologies are not able to prevent the spread of viruses and antibiotic resistance is becoming more prevalent [8-10]. Patients in the ICU and immunocompromised patients are particularly susceptible to airborne infectious agents and these infections may be lethal [3, 6]. Additionally, patients undergoing surgical procedures may be at higher risk for complications due to pathogenic contamination of the air [11-12]. 

While it is known that the indoor air environment plays a critical role in the health of patients in hospitals and buildings, more stringent air quality standards and standardized monitoring protocols in the hospital setting are needed [1]. Previous studies with portable air purification in the hospital setting have shown the potential to reduce potentially infectious bacteria and mold and have stressed the importance of patients breathing clean air in their local environment [13-15]. Ours is the first report to our knowledge showing how this may impact health outcomes such as length of stay. 

Major problems with current mechanical air filtration, even using high-efficiency particulate air filters and the HVAC systems used in hospitals, include the ability of micro-organisms to proliferate on the filter surfaces themselves and the inability for existing technology to effectively stop the spread of small particles such as viruses and other toxins [16-17].

With the portable PECO units, in addition to physical filtration, a photo-electrochemical reaction takes place on the surface of a nano-coated filter leading to the oxidation of organic matter. These processes allow for the destruction of organic material 1000 times smaller than what a traditional filter can capture [7]. Thus, PECO can efficiently destroy airborne organic matter, bacteria, viruses, mold, and volatile organic compounds converting them into their trace elements [7].

There are a few limitations to the study. This study was designed as a pre-post intervention trial and the findings can be associated with the phenomenon of “regression to the mean”. However, we consider the 54% decline in the use of non-invasive ventilation use as impactful from a health systems perspective and although not statistically significant, the results were consistent in terms of decline for all outcomes thereby providing confidence in the estimates associated with the efficacy of the air purification device. Of note, this trend was also observed in the adjusted analysis as well as using the propensity score. Furthermore, a follow of 5 months would have potentially demonstrated an upward or downward trend to confirm the regression to the mean phenomenon which we could not detect. However, we do acknowledge that a prospective cluster-randomized trial would be the most unbiased study to provide a conclusive answer to the question posed in this study but this may not be possible due to logistical and ethical considerations. Nevertheless, despite the limitations, these findings provide the first-ever hypothesis-generating findings related to the efficacy of an air filtration device to reduce resource use in the pediatric ward setting. These findings will also inform the design and conduct of future studies that can address potentially unknown confounders as the groups were balanced for known confounders in the current study.

## Conclusions

In conclusion, portable PECO air purification appears to reduce hospital stay and need for invasive or non-invasive intervention for pediatric patients admitted with respiratory distress. This is an important clinical outcome that could provide significant healthcare provider cost savings and deserves a larger-scale study. In addition, it was found that possible pathogens in the patient’s local environment may be effectively removed from the air with this technology. 

## Figures and Tables

**Figure 5:**
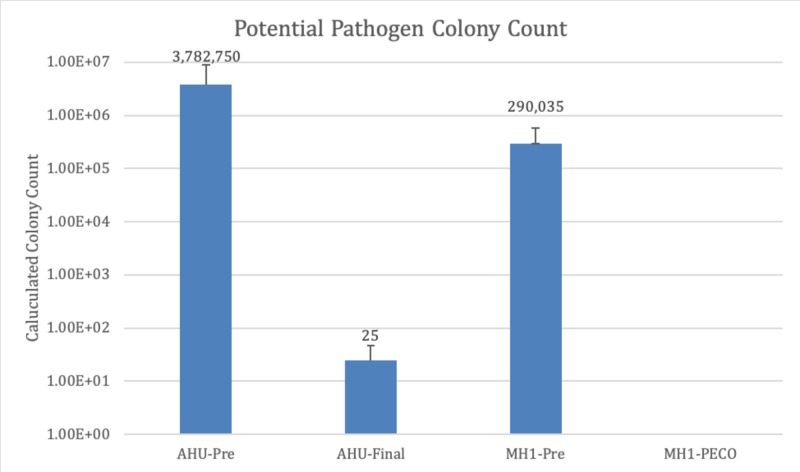
Pre and final filter bacterial count AHU versus PECO portable unit (MH1) AHU, air handling unit; PECO, photo-electrochemical oxidation

**Figure 6:**
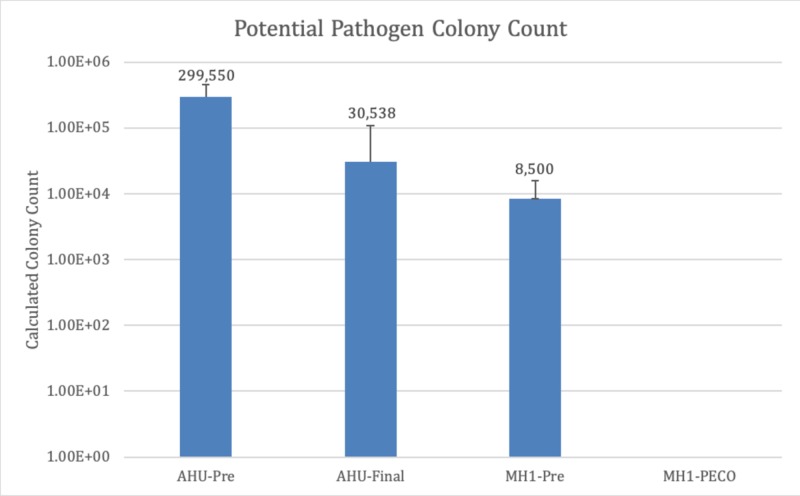
Pre and final filter fungal counts AHU versus PECO portable unit (MH1) AHU, air handling unit; PECO, photo-electrochemical oxidation

## Appendices

Microbial sampling of the portable PECO pre-filter and final filters, as well as sampling of the HVAC MERV-rated central air handling units (AHU) pre-filter and final filter, was assessed twice during the study. Filter samples were collected by trained laboratory professionals who swabbed the surfaces, preserved them on ice and sent them to an outside lab (EMSL Analytical, Inc) for microbial analysis. Swabbing was conducted using sterile swabs, which were wetted and run along the length of the pleats of the filters. Following collection, swabs were promptly placed within the sterile collection tube and sealed. Collection for the AHU-Pre involved collecting from two pleats and the AHU-Final involved collecting from 1 single pleat, the reasoning for 1-1-pleat collection was due to the AHU-Final filter’s large surface area. Collection for the MH1- Pre and MH1-PECO both involved collecting from 4 pleats to increase coverage of the area sampled. Prefilter and final filters were assessed for microbial pathogens including bacteria and fungi from at least 3 different pleats to best represent each filter as a whole and samples were averaged.  The results are shown in figure 5 and figure 6 below.  

## References

[1] Shajahan A, Culp CH, Williamson B (2019). Effects of indoor environmental parameters related to building heating, ventilation, and air conditioning systems on patients' medical outcomes: a review of scientific research on hospital buildings. Indoor Air.

[2] Heutte N, Andre V, Dubos Arvis C (2017). Assessment of multi-contaminant exposure in a cancer treatment center: a 2-year monitoring of molds, mycotoxins, endotoxins, and glucans in bioaerosols. Environ Monit Assess.

[3] Demuyser T, De Cock E, Sermijn E (2019). Airborne Aspergillus fumigatus contamination in an intensive care unit: detection, management and control. J Infect Public Health.

[4] Xiao S, Tang JW, Hui DS, Lei H, Yu H, Li Y (2018). Probable transmission routes of the influenza virus in a nosocomial outbreak. Epidemiol Infect.

[5] Sublett JL, Seltzer J, Burkhead R, Williams PB, Wedner HJ, Phipatanakul W (2010). Air filters and air cleaners: rostrum by the American Academy of Allergy, Asthma & Immunology Indoor Allergen Committee. J Allergy Clin Immunol.

[6] Holy O, Matouskova I, Kubatova A, Hamal P, Svobodova L, Juraskova E, Raida L (2015). Monitoring of microscopic filamentous fungi in indoor air of transplant unit. Cent Eur J Public Health.

[7] Rao NG, Kumar A, Wong JS, Shridhar R, Goswami DY (2018). Effect of a novel photoelectrochemical oxidation air purifier on nasal and ocular allergy symptoms. Allergy Rhinol.

[8] Mirhoseini SH, Nikaeen M, Khanahmad H, Hassanzadeh A (2016). Occurrence of airborne vancomycin- and gentamicin-resistant bacteria in various hospital wards in Isfahan, Iran. Adv Biomed Res.

[9] Spivak ES, Hanson KE (2018). Candida auris: an Emerging Fungal Pathogen. J Clin Microbiol.

[10] Tellier R, Li Y, Cowling BJ, Tang JW (2019). Recognition of aerosol transmission of infectious agents: a commentary. BMC Infect Dis.

[11] Cook TM, Piatt CJ, Barnes S, Edmiston CE, Jr Jr (2019). The impact of supplemental intraoperative air decontamination on the outcome of total. Joint Arthroplasty: A Pilot Analysis. J Arthroplasty.

[12] Dehghani M, Sorooshian A, Nazmara S, Baghani AN, Delikhoon M (2018). Concentration and type of bioaerosols before and after conventional disinfection and sterilization procedures inside hospital operating rooms. Ecotoxicol Environ Saf.

[13] Abdul Salam ZH, Karlin RB, Ling ML, Yang KS (2010). The impact of portable high-efficiency particulate air filters on the incidence of invasive aspergillosis in a large acute tertiary-care hospital. Am J Infect Control.

[14] Boswell TC, Fox PC (2006). Reduction in MRSA environmental contamination with a portable HEPA-filtration unit. J Hosp Infect.

[15] Pantelic J, Sze-To GN, Tham KW, Chao CY, Khoo YC (2009). Personalized ventilation as a control measure for airborne transmissible disease spread. J R Soc Interface.

[16] Kim SH, Ahn GR, Son SY, Bae GN, Yun YH (2014). Mold occurring on the air cleaner high-efficiency particulate air filters used in the houses of child patients with atopic dermatitis. Mycobiology.

[17] Price DL, Simmons RB, Crow SA, Jr. Jr., Ahearn DG (2005). Mold colonization during use of preservative-treated and untreated air filters, including HEPA filters from hospitals and commercial locations over an 8-year period (1996-2003). J Ind Microbiol Biotechnol.

